# Daily fecal pH pattern and variation in lactating dairy cows

**DOI:** 10.3168/jdsc.2021-0158

**Published:** 2022-02-10

**Authors:** Rafael Alejandro Palladino, María Florencia Olmeda, Nicolás Juliano, Fernando Bargo, Ignacio R. Ipharraguerre

**Affiliations:** 1 CONICET, Ruta 4 y Juan XXIII, Lavallol 1832, Argentina; 2 Facultad de Ciencias Agrarias, Universidad Nacional de Lomas de Zamora, Ruta 4 y Juan XXIII, Lavallol 1832, Argentina; 3 Fundación Instituto de la Leche, Ruta 205 km 51, Paraje Estancia San Martín, Cañuelas 1814, Argentina; 4 Facultad de Agronomía, Universidad de Buenos Aires, Av San Martín 4453, Ciudad Autónoma de Buenos Aires 1417, Argentina; 5 Institute of Human Nutrition and Food Science, University of Kiel, Kiel 24118, Germany

## Abstract

Evidence supports a causal link between anomalous intestinal function and impaired performance in dairy cows. Consequently, digesta pH values obtained from colon, cecum, and rectum are increasingly used to monitor intestinal function in dairy cows. We conducted a study to describe the daily dynamics of fecal pH in lactating dairy cows. The study lasted 4 d and individual records of dry matter intake, milk yield, and fecal pH were taken. Samples of feces were taken every 4 h during the 4-d study, and sampling time was adjusted ahead by 1 h daily so that a sample was obtained for each 1-h interval of the day. Data were analyzed using a mixed-effect model including time as fixed effect and cow as a random factor. We performed a cosinor analysis using pH data at different time points to determine whether fecal pH followed a biorhythmic pattern. On average, cows consumed 19.1 ± 1.55 kg/d of dry matter and produced 26.3 ± 4.16 kg/d of milk. The most relevant results confirmed a biorhythmic pattern for feces pH around feeding time: mesor (midline estimating statistic of rhythm) 6.20, amplitude 0.28, and acrophase 5.66. Additionally, we found a positive relationship between dry matter intake and amplitude, possibly because of an increase in the amount of fermentable carbohydrate reaching the hindgut in response to increasing intake. When using fecal pH as an indicator of intestinal function, it is critical to obtain samples at several time points to capture its daily rhythmicity and to report sampling time relative to feeding.

## Highlights

• Fecal pH in healthy dairy cows follows a biorhythmic pattern.
• Fecal pH mesor (daily mean) occurs around feeding time.
• Fecal pH amplitude (distance from mean to peak) is positively correlated with dry matter intake.

## Graphical Abstract

**Figure:**
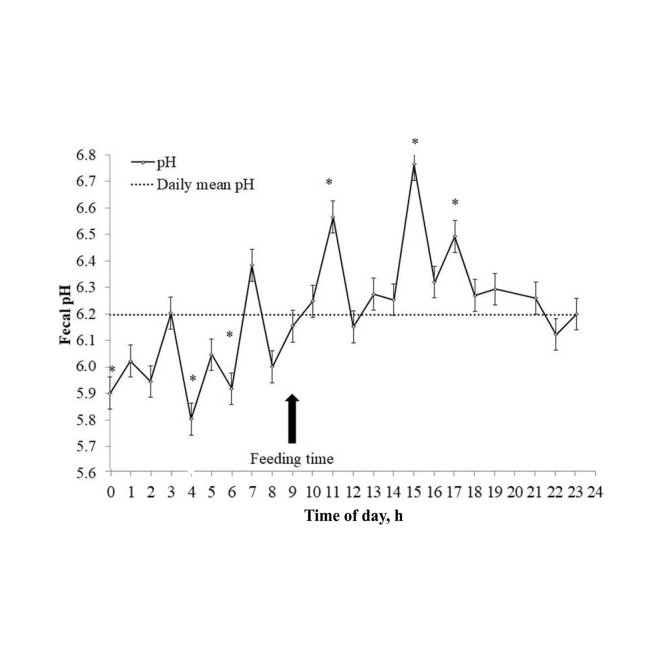
**Summary:** The daily dynamics of fecal pH in healthy dairy cows is currently unclear. In view of the diagnostic value and growing research interest in this trait as a surrogate indicator of intestinal function, we aimed to describe the daily pattern of fecal pH in lactating cows fed once a day. Fecal pH pattern varied throughout the day, showing a mean value around feeding time. More importantly, fecal pH showed a harmonic pattern, with mesor (daily mean) occurring around feeding time and amplitude (distance from mean to peak) positively correlated with DMI. Therefore, when using feces to investigate the effect of dietary, management, or other factors on the pathological alteration of fecal pH and its correlation with intestinal physiology and gut microbiota in dairy cows, it is recommended to obtain samples and report readouts at several time points to capture the daily rhythmicity of fecal pH.

A growing body of evidence supports a causal link between anomalous intestinal function and impaired productivity and health in dairy cows. Even though fermentation in the large intestine is a normal process that supplies energy (VFA) to cows (Bergman, 1990), the ruminal outflow of fermentable carbohydrates can sometimes reach disproportionate levels, eventually provoking intraluminal accumulation of organic acids (Plaizier et al., 2008; Gressley et al., 2011) and perturbation of the intestinal microbiota (Plaizier et al., 2017). The consequential increase in large intestinal acidity and osmolarity not only hampers fermentation and nutrient uptake (Holtug et al., 1992; Plaizier et al., 2008) but it may also override mucosal defense mechanisms, causing pathological secretion of mucus and fluids (diarrhea) (Plaizier et al., 2008), enhanced turnover of epithelial cells (Saunders and Sillery, 1982), and defective functioning of the intestinal barrier (Aschenbach et al., 2019). These physiological alterations are commonly paralleled by proliferation of *Enterobacteriaceae*, loss of commensal bacteria, and buildup of microbial-associated molecular patterns, such as LPS, in the hindgut (Li et al., 2012; Plaizier et al., 2017). Collectively, these factors increase the potential for intestinal and systemic inflammation, limit the availability of nutrients for productive purposes, and compromise animal welfare (Gressley et al., 2011; Aschenbach et al., 2019). Paradoxically, high-producing animals that must consume large amounts of digestible feeds to meet their nutritional needs are at a higher risk of suffering such ailments. Furthermore, several factors other than diet-related causes can induce pathophysiological alterations in the intestinal mucosa accompanied by dysbiosis (Levy et al., 2017). Some of those etiological factors, such as inflammation (Zeng et al., 2017) and hyperthermia (Koch et al., 2019), are indeed major challenges in modern dairy production. Considering these ideas, the pH of digesta obtained from the colon, cecum, or rectum (feces) is increasingly used to monitor intestinal function in dairy cows.

Previous work with sheep and steers showed that feces have similar pH to the contents of the small intestine and colon (Wheeler and Noller, 1977), supporting the use of fecal pH as a valid surrogate of intestinal pH in ruminants. Consistent with this hypothesis, several published studies have relied on fecal pH to examine the effect of diverse dietary interventions on the intestinal function of dairy cows (Clayton et al., 1999; Osborne et al., 2004; Li et al., 2012; Danscher et al., 2015; Sulzberger et al., 2016; Meyer et al., 2019; Petri et al., 2019). In all cases, pH was measured in either one or a few time-spaced fecal samples, and information about the sampling time relative to the time of feed delivery was often not provided. Much like in the rumen, however, microbial activity in the large intestine is governed by environmental factors (e.g., substrate availability) that are prone to changes in daily rhythm (Bergman, 1990; Gressley et al., 2011). For instance, the concentration of NDF in feces of dairy cows follows a 24-h pattern that is entrained by feeding time (Niu et al., 2014), suggesting that the availability of fermentable substrates in the lower gut presents similar rhythmicity. In addition, epithelial cells lining the intestinal walls react to changes in microbial activity by adjusting their diurnal metabolic rhythms (Kuang et al., 2019) and the secretion of compounds such as bicarbonate, mucins, and antimicrobial peptides that profoundly modify the luminal environment (Gressley et al., 2011; Pardo-Camacho et al., 2018). Therefore, it seems reasonable to expect that the pH in the large intestinal lumen (and feces) varies throughout the day, showing distinct patterns in response to factors such as diet, feeding management, and stress.

In view of the diagnostic value and growing research interest in fecal pH, we conducted this study to describe the daily dynamics of fecal pH in lactating cows. Twelve Holstein lactating cows (541 ± 57.1 kg of BW, 110 ± 8 DIM, BCS of 3.0 ± 0.29, lactation 1.6 ± 1) were allocated in individual pens (pen size was 5 m × 13 m) with individual feeders and waterers at the Dairy Research Farm of Fundación Instituto de la Leche (Suipacha, Argentina; www.fil.org.ar). All procedures involving cows were approved by the Ethics Animal Commission of the Facultad de Ciencias Agrarias, Universidad Nacional de Lomas de Zamora, Argentina. The study lasted 4 d, during which time measurements of individual DMI, milk yield (**MY**), and fecal pH were taken. Cows were milked twice daily at 0500 and 1700 h and individually offered a TMR at 0900 h (once a day) at a rate of 110% of previous daily intake (as-fed basis). The TMR was prepared before feeding and was composed of corn silage (50%), ground corn (19.5%), wheat bran (17.5%), extruded soybean meal (11.15%), and a vitamin-mineral premix (1.85%; Provimi, Cargill Argentina) containing 21% Ca, 2% P, 3% Mg, 3% Na, 4.4% Cl, 900 mg/kg Cu, 3,600 mg/kg Zn, 2,500 mg/kg Mn, 30 mg/kg Se, 50 mg/kg I, 150,000 IU of vitamin A, 60,000 IU of vitamin D_3_, 250 IU of vitamin E, and 1,200 mg/kg monensin. Feed refusals were collected every day at 0800 h and used to calculate DMI as the difference between feed offered and refused. Corn silage [DM 27.0%, ash 5.2% CP 7.6%, ether extract (**EE**) 2.5%, NDF 41.1%, ADF 18.4%, and starch 27.7%] and concentrate (DM 89.4%, ash 8.0% CP 19.4%, EE 5.1%, NDF 23.1%, ADF 8.6%, and starch 36.9%) were sampled before starting the experiment, and TMR (DM 42.9%, ash 6.7% CP 14.1%, EE 3.9%, NDF 31.6%, ADF 13.2%, and starch 32.6%) samples were taken daily for composition analyses (DM, CP, EE, and ash by AOAC International, 2000; NDF and ADF by Van Soest et al., 1991). Milk yield was recorded daily and individual milk samples were taken for milk composition analyses. Fat, total protein, and lactose were determined by mid-infrared analysis (MilkoScan FT6000, Foss Analytical A/S). Samples of feces were taken every 4 h during the 4-d study. The sampling time was adjusted ahead by 1 h daily so that a sample was obtained for each 1-h interval of the day (24 total samples). Fresh feces were taken directly from the rectum of each cow; 50 ± 1 g was weighed using a digital scale (model SF-400, Gromy Industry Co. Ltd.) and placed into a plastic jar with screw cap. The jar was immediately filled with distilled water to complete 100 g and inverted 20 times until the feces were suspended. Subsequently, fecal pH was measured using a glass electrode (Hanna Instruments).

After checking residuals for outliers and confirming normality, data were analyzed using a mixed-effect model (SAS Institute, 1996) and repeated-measures analyses including time as fixed effect and cow as a random factor. Covariance structures were tested, and selection was done based on Bayesian information criterion. The PDIFF statement of SAS (SAS Institute, 1996) was used to compare means at different time points. Statistical differences were declared when *P* < 0.05 and trends were discussed at *P* < 0.10. Additionally, a cosinor analysis was performed using pH data at different time points to determine whether fecal pH follows a biorhythmic pattern. Amplitude, time between peaks (period), and time at peak (phase) for fecal pH were determined using a linear form of the cosine function with a 24-h period. The original model included the mesor (midline estimating statistic of rhythms; mean pH) and the harmonic terms for 4.8, 8, and 12 h. Based on the Bayesian information criterion, reduction of the model excluded all the harmonic coefficients. The phase was reported as acrophase, which is the time when the peak of a rhythm occurs (Bourdon et al., 1995). Finally, the relationship between fecal pH, production traits (DMI, MY), and biorhythm coefficients (mesor, amplitude, acrophase) were assessed using Pearson correlation (SAS Institute, 1996).

On average, cows consumed 19.1 ± 1.55 kg/d of DM and produced 26.3 ± 4.16 kg/d of milk with 3.29 ± 0.448% fat, 3.10 ± 0.217% protein, and 4.72 ± 0.124% lactose. Although variable, fecal pH showed a distinct 24-h pattern characterized by an upward trend starting at a nadir (5.80) at 0400 h, peaking (6.76) at 1500 h, and dropping rapidly in the following 3 h to a level between 6.27 and 5.95 (Figure 1). Across the 24 h, fecal pH averaged 6.20 ± 0.091 and differed (*P* < 0.05) from values registered at 0000, 0400, 0600, 1100, 1500, and 1700 h. These hourly values exceeded (*P* < 0.05) the daily mean only within the 8-h interval that followed TMR feeding (6.57, 6.76, and 6.48 at 1100, 1500, and 1700 h, respectively). Thereafter, they became progressively lower than the daily mean, reaching significance (*P* < 0.05) at 15, 19, and 21 h post-feeding (5.90, 5.80, and 5.92 at 0000, 0400, and 0600 h, respectively). When pH data were modeled using a cosinor function (Figure 2), a biorhythmic pattern was confirmed with the following parameters: mesor 6.20, amplitude 0.28, and acrophase 5.66. Correlation analysis revealed that no significant relationship existed between daily mean fecal pH (or mesor) and MY (*P* > 0.05). However, a positive relationship was found between DMI and amplitude (r = 0.67; *P* < 0.05). Additionally, the negative correlation between DMI and pH at 4 h was significant (r = −0.71; *P* < 0.01). As previously mentioned, pH at 4 h was the lowest pH throughout the day. The average acrophase coincided with the time at which the highest pH values were recorded (pH at 1500 h was approximately 5 h after pH at 1000 h, the last time point that was nonsignificantly different from mean pH).

**Figure 1:**
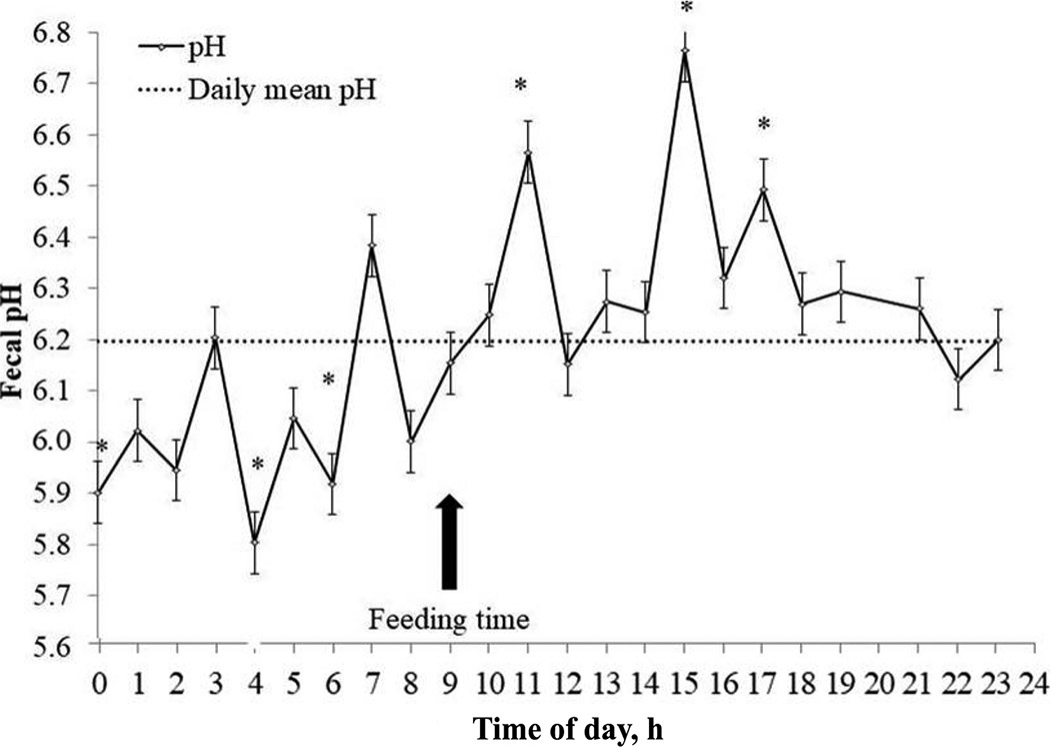
Daily fecal pH pattern in well-fed lactating dairy cows. Mean (±SE) comparison corresponds to hourly pH versus daily mean fecal pH (*P < 0.05).

**Figure 2:**
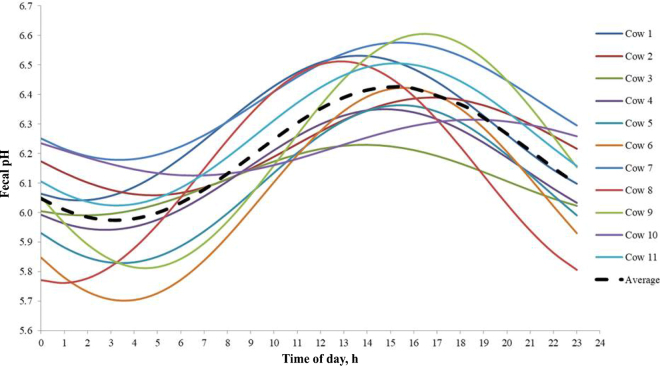
Daily fecal pH pattern for individual cows used in the experiment modeled by the cosinor function. Feeding time was at 0900 h.

To best of our knowledge, previous studies focusing on the daily pattern of fecal pH in dairy cows were conducted under the context of experimentally induced ruminal acidosis (Osborne et al., 2004; Danscher et al., 2015; Sulsberger et al., 2016), which could misrepresent the intestinal physiology of well-managed, healthy dairy cows. Therefore, we decided to examine the daily rhythmicity of such a trait in dairy cows fed and managed according to current standards of dairy production. Van Baale et al. (2004) studied the effect of forage or grain-based diets and monensin inclusion on ruminal and fecal persistence of *Escherichia coli* O157:H7. Fecal pH measured in samples taken 3 h after feeding was not affected when the inclusion of monensin was evaluated. According to our results, the likelihood of finding significant differences between diets at 3 h after feeding is low. Other studies measuring fecal pH did not report the time at which samples were taken (Clayton et al., 1999; Li et al., 2012; Meyer et al., 2019; Petri et al., 2019), which, considering our results, is a major hindrance to study-wise comparisons. Even treatment-wise comparisons within those studies are questionable because we showed that performance variables were not related to mean fecal pH, whereas DMI was negatively correlated with fecal pH at 5 h before feeding time (0400 h) and positively correlated with amplitude. Gressley et al. (2011) suggested that high-producing animals are at a higher risk of hindgut acidosis because of a greater amount of rapidly fermentable carbohydrates reaching the large intestine. Our results show that amplitude seems to be a better indicator of hindgut fermentation rather than a single pH value or even mean daily pH. It seems reasonable to propose, therefore, that studies aiming to use fecal pH as an indicator of intestinal fermentation or dysbiosis should report daily patterns and, preferably, parameters of a cosinor function to better describe hindgut physiology. However, more research is needed to establish the minimum number of samples required to obtain reliable estimates of biorhythm parameters and their association with biomarkers of intestinal function, microbiota composition, and microbial activity. Furthermore, it seems warranted to investigate whether a threshold amplitude and time elapsed under such a threshold correlate with intestinal dysfunction.

In conclusion, fecal pH showed a harmonic pattern with a mesor pH occurring around feeding time. Amplitude was positively correlated with DMI, possibly because of an increase in the amount of fermentable carbohydrate reaching the hindgut in response to increased intake. When the aim is to use fecal pH as a surrogate indicator of intestinal function, it is critical to obtain samples at several time points to capture its daily rhythmicity and to report sampling time relative to feeding. Further research is needed to gain a better understanding of how factors such as feeding management can cause pathological entrainment of fecal pH and its correlation with intestinal physiology and gut microbiota.
